# ISAMBARD: an open-source computational environment for biomolecular analysis, modelling and design

**DOI:** 10.1093/bioinformatics/btx352

**Published:** 2017-06-05

**Authors:** Christopher W Wood, Jack W Heal, Andrew R Thomson, Gail J Bartlett, Amaurys Á Ibarra, R Leo Brady, Richard B Sessions, Derek N Woolfson

**Affiliations:** 1 School of Chemistry, University of Bristol, Bristol, UK; 2 School of Biochemistry, University of Bristol, Bristol, UK; 3 School of Chemistry, University of Glasgow, Glasgow, UK; 4 BrisSynBio, University of Bristol, Bristol, UK

## Abstract

**Motivation:**

The rational design of biomolecules is becoming a reality. However, further computational tools are needed to facilitate and accelerate this, and to make it accessible to more users.

**Results:**

Here we introduce ISAMBARD, a tool for structural analysis, model building and rational design of biomolecules. ISAMBARD is open-source, modular, computationally scalable and intuitive to use. These features allow non-experts to explore biomolecular design *in silico*. ISAMBARD addresses a standing issue in protein design, namely, how to introduce backbone variability in a controlled manner. This is achieved through the generalization of tools for parametric modelling, describing the overall shape of proteins geometrically, and without input from experimentally determined structures. This will allow backbone conformations for entire folds and assemblies not observed in nature to be generated *de novo*, that is, to access the ‘dark matter of protein-fold space’. We anticipate that ISAMBARD will find broad applications in biomolecular design, biotechnology and synthetic biology.

**Availability and implementation:**

A current stable build can be downloaded from the python package index (https://pypi.python.org/pypi/isambard/) with development builds available on GitHub (https://github.com/woolfson-group/) along with documentation, tutorial material and all the scripts used to generate the data described in this paper.

**Supplementary information:**

Supplementary data are available at *Bioinformatics* online.

## 1 Introduction

Generally, the three-dimensional structures of biomolecules determine their functions. The computational design of such structures—and proteins in particular—tests and advances our understanding of biomolecular folding and assembly, and paves the way to constructing entirely new biomolecules with applications in biotechnology and synthetic biology. Here we present a new suite of computational tools, which we call ISAMBARD (Intelligent System for Analysis, Model Building And Rational Design), to aid the rational *de novo* design of biomolecular structures and assemblies, and for the *in silico* assessment of the resulting design models. The overall aims of ISAMBARD are to provide easy-to-use tools for the parametric design of such structures, and, thus, to enable a wider group of both expert and non-expert computational and experimental users to engage in the design process.

Several approaches are taken in protein design (Huang *et al.*, 2016; Porebski and Buckle, 2016; Regan *et al.*, 2015; Woolfson *et al.*, 2015): In *protein redesign*, natural proteins are used as starting points and engineered to introduce desired structural, stability, or functional properties. This is guided intuitively, or, increasingly, computationally. In *rational de novo* protein design, chemical and physical principles, and biochemical rules of thumb for protein folding are combined to make initial designs, which are improved by iteration. In *computational design*, *de novo* sequences are built *in silico* onto protein backbones, which can be static or have some flexibility, to deliver multiple sequences for experimental testing.

A number of approaches to computational protein design have yielded success (Huang *et al.*,2016; MacDonald and Freemont, 2016; Woolfson *et al.*, 2015). Initial efforts involved sequence-based redesign, where designs are generated by packing new sequences onto a backbone scaffold from a known protein structure (Dahiyat and Mayo, 1997). Building on this, a degree of backbone flexibility can be introduced using fragment-based design, where regions of known protein structure are combined together to form new backbone models. The most successful implementation of this method is in Rosetta (Das and Baker, 2008), a macromolecular modelling package, which has been central to many *de novo* designs including the novel fold Top7 and, more recently, *de novo* repeat proteins (Doyle *et al.*, 2015; Kuhlman *et al.*, 2003). Extensions of the fragment-based methodology are being actively developed (Jacobs *et al.*, 2016; Lapidoth *et al.*, 2015).

By definition, fragment-based methods are restricted, sampling only structural space observed in experimentally determined, and usually of just natural protein structures. If we are to exploit the full universe of possible protein structures, other backbone sampling methods must be pursued (Taylor *et al.*, 2009; Woolfson *et al.*, 2015). However, there are several obstacles in the way of achieving this. Most notably, the sequence and conformational spaces available to even modestly sized biomolecules are vast, and, indeed, impossible to search exhaustively. One way to reduce this complexity is to simplify the way in which biomolecular structures are described; namely, to parameterize the design target mathematically. In turn, these parametric descriptors can be used to focus the search of structural space for the backbone. Amino-acid sequences can then be tested on the resulting scaffolds, and the whole system optimized to deliver candidate solutions to a specified design problem.

Certain folds are conducive to parameterization, such as α-helical coiled coils, due to their regular structures and well-understood sequence-to-structure relationships (Fletcher *et al.*, 2012; Harbury *et al.*, 1993, 1994; Woolfson *et al.*, 2012; Woolfson, 2005). α-Helical coiled coils are bundles of two or more α helices that invariably wrap (or supercoil) around a common axis. The helices can be arranged in parallel, antiparallel or mixed topologies, and the assemblies can be homo- or hetero-oligomers (Lupas and Gruber, 2005). Despite this diversity, α-helical coiled coils are the simplest and best-understood examples of geometrically regular protein structures, making them clear targets for parametric modelling and design. The original mathematical parameterization of these is from Crick (Crick, 1953), and has been developed since (Offer *et al.*, 2002), including in CCCP (Grigoryan and Degrado, 2011) and CCBuilder (Wood *et al.*, 2014), which are web-based applications for parametric modelling of coiled coils. These modelling methods have been applied by us and by others to design a range of α-helical coiled coils and bundles (Grigoryan *et al.*, 2011; Harbury *et al.*, 1995; Huang *et al.*, 2014; Thomson *et al.*, 2014).

The structural modelling methodology that we have applied to design α-helical barrels required an extension of CCBuilder, called CCScanner, which automatically fitted structural parameters for a given sequence (Thomson *et al.*, 2014). However, this was a bespoke solution for the parametric modelling of coiled coils. Here, we present the ISAMBARD (Intelligent System for Analysis, Model Building and Rational Design) software package, which generalizes this modelling methodology, allowing it to be applied to the design of any parameterizable protein fold, whether all-α helix, all-β strand, mixed α/β structures, or those employing less-common secondary structures. ISAMBARD is an open-source Python package with a suite of tools for biomolecular structure analysis, protein design, model building and evaluation. ISAMBARD is modular, extendable, open source and freely available.

ISAMBARD provides a framework for atomistic model building and validation of truly *de novo* biomolecular structures (Woolfson *et al.*, 2015). Scoring methods are built-in for assessing model quality, and optimization techniques allow rapid exploration of structural and sequence space in tractable time. Here, we demonstrate that ISAMBARD is capable of accurately modelling a range of diverse protein folds using generalized and reusable mathematical parameterizations.

## 2 Materials and methods

All biomolecules in ISAMBARD are represented using the AMPAL (Atom, Monomer, Polymer, Assembly, Ligand) framework. This is a formal representation of biomolecules in a hierarchical structure of lightweight Python objects. Its object-oriented implementation is intuitive to use and enables facile navigation through the protein structure in both directions, i.e. from the assembly to the atomic level and *vice versa*.

AMPAL objects are used in ISAMBARD to represent proteins, nucleic acids, and a more-general ligand class that is currently used for every other molecule. There are a range of tools built into these objects, which allows for straightforward structural analysis, validation and manipulation.

**Fig. 1.**
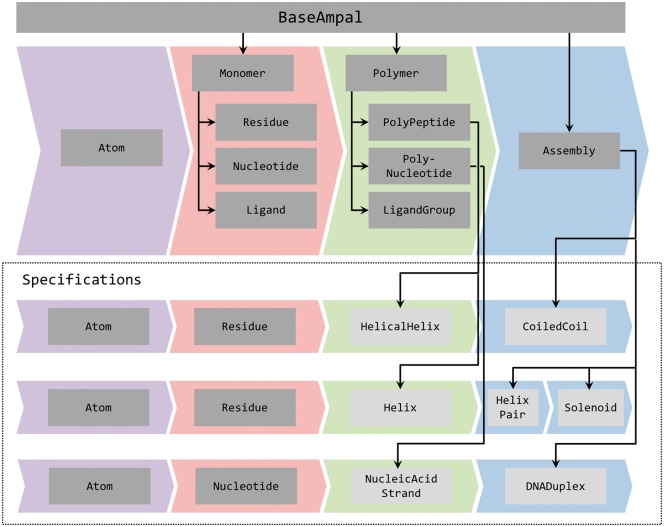
Inheritance in the AMPAL framework. Top: Arrows indicate inheritance, with objects at the head of the arrow inheriting all of the methods and attributes of the more generic object at the base of the arrow. Bottom: Examples of specifications in the AMPAL framework. The specification classes are shown in light grey boxes

Figure 1 shows the structure of the AMPAL framework and its built-in inheritance pattern. This enables core functionality to be reused, making it simpler for users to create custom classes for other biomolecules.

### 2.1 Parametric model building

ISAMBARD has been created to aid parametric protein design by providing a general approach for modelling any parameterizable protein fold. In order to design protein folds *de novo*, one must choose from a set of amino acids and connect them in space according to a set of rules, in an approach analogous to that followed by a building constructor using an architect’s design or specification. Therefore, we have introduced the specification object, as an extension of the AMPAL framework (Fig. 1). A specification contains instructions for building a model according to a set of input parameter values. These instructions form the parameterization of the model. Specifications can be defined at both the Polymer and Assembly level of the AMPAL framework (monospaced text indicates an ISAMBARD class). The parameters in Polymer specifications dictate how to arrange Monomers into a single chain; at the Assembly level, they detail the arrangement of Polymers with respect to each other.

#### 2.1.1 Specifications at the polymer level

Each Residue in a Polypeptide contains an α-carbon atom, and the running average of the positions of these atoms traces a path in 3D space. Polypeptide specifications use parameters that define a path for this running average to follow. When the model is built, Residues are joined together accordingly. The paths, and therefore the Polypeptides, are described mathematically by a small number of simple parameters. For example, the Helix specification allows any type of polypeptide helix to be built, e.g. α helix, polyproline type-II helix, etc.; whereas, the HelicalHelix specification takes a Helix specification and adds a supercoil to it with input parameter values for radius and pitch of the superhelix. In this way, a path is defined along which a polypeptide segment is built. Moreover, multiple segments with different Helix and HelicalHelix specifications can readily be combined in the same design (see the Assembly specification below). As indicated, these specifications are implemented generally, such that secondary structure types including α-, collagen- (viz., polyproline type-II-) and π-helices can be built along any well-defined path. It is worth reemphasizing at this point that these parameters are not reliant on structural data from natural proteins, they are built using idealized geometric models.

An alternative building-mode specification is embodied in TAPolypeptide, which generates a Polypeptide from a set of backbone torsion angles. Backbone bond lengths and bond angles can be specified if desired, otherwise default values are used (Schulz and Schirmer, 1979). Again, this lends itself to the design of structures that are not found in nature, but, nonetheless, are physically feasible, as they can be informed by the allowed regions of Ramachandran plots.

#### 2.1.2 Specifications at the assembly level

Specifications at the Assembly level are relatively abstract, and are not constrained to describing a particular protein topology, architecture or even class. Three examples of specifications at the Assembly level are given in Supplementary Figures S1–S4. They describe the paths that secondary structure follows, and the same specification can be used to describe a range of folds. For example, the CoiledCoil specification can produce models of coiled coils in any oligomer state with any orientation of helices. Furthermore, the same specification can be used to describe the structure of the collagen triple helix.

Up to this point, the building process uses glycine as default residues, essentially generating a backbone-only model. Once this backbone for the target structure has been specified, side-chain atoms are modelled using SCWRL4 (Krivov *et al*, 2009), which uses a backbone-dependent rotamer library and a fast anisotropic hydrogen bonding function to optimize side-chain packing.

#### 2.1.3 Model evaluation

The main method for assessing the quality of the model uses BUFF (Bristol University Docking Engine Force Field). BUFF is a stand-alone implementation of the all-atom force field from BUDE (Bristol University Docking Engine) (McIntosh-Smith *et al.*, 2012, 2014), which is an empirical free-energy force field originally designed to predict the free energies of binding between proteins and ligands.

BUFF is implemented with code written in C ++ and Python, with communication between these achieved by a layer of Cython (Behnel *et al.*, 2011). The Cython layer allows for direct interaction with various elements of the force field using a Python interface, which is useful when prototyping design protocols, but it retains most of the speed of the original BUDE implementation. This also allows the force-field parameters to be directly accessible to the user, and modifiable for a particular application.

Other metrics are also available for assessing design quality, such as evaluating the overall geometry of the protein; for example, we have included a measure of helical strain, which assesses how far from ideal geometry a helix undergoing design is. Moreover, the modular and open nature of ISAMBARD enables and encourages users to import and apply other force fields and methods of evaluation. This is facilitated by the Python ecosystem in general, which contains a range of existing packages for protein design and modelling, such as OpenMM, PyRosetta and Modeller (Chaudhury *et al.*, 2010; Eastman *et al.*, 2013; Eswar *et al.*, 2006).

#### 2.1.4 Parameter optimization

The size of structural space grows exponentially with the number of parameters used to describe it. This prohibits the exhaustive exploration of space in most cases. So-called metaheuristics help address this, providing means of efficiently searching the defined parameter space to find near-optimal solutions (Bianchi *et al.*, 2008). A range of metaheuristics have been implemented in ISAMBARD using modified elements of the DEAP evolutionary computation framework (Fortin *et al.*, 2012), including a genetic algorithm, particle-swarm optimization, differential evolution and covariance matrix adaptation evolutionary strategy. These different methods enable efficient exploration of structural space for a given specification and provide an estimate of energetic minima.

The choice of optimizer is up to the user: different optimizers will be better suited to different problems. For the examples described herein, we found that the differential evolution method performed very well. Further work to benchmark each of the optimization strategies is underway in our laboratory, and is beyond the scope of this report.

Once a fold has been parameterized, minimal human intervention is required: the optimizer fits a broad range of parameter values from the specification and delivers the best models according to the user-defined fitness function. For protein design, this is usually an all-atom scoring function, but any metric can be applied by the user.

### 2.2 Specification accuracy testing

To test the robustness of models produced using ISAMBARD, several protein folds were parameterized. The geometric parameterizations were tested by rebuilding natural structures that exhibited a wide range of parameters. During the rebuild, we used the root-mean-square deviation (RMSD) between the experimentally determined structure and models produced to drive the parameter optimization. This process validates whether the simple geometric parameterization has the capacity to recreate accurately observed examples of the protein fold, and thus lends confidence to modelling *de novo* structures. Three classes of protein were modelled: α-helical coiled coils, collagen/collagen-like peptides, and Ankyrin-like repeat proteins.

We used the differential evolution optimizer in ISAMBARD to fit the parameters for a given sequence. The scoring metric used was the RMSD between the target structure and the model as calculated by the McLachlan algorithm (McLachlan, 1982) as implemented in the program ProFit (Martin, A.C.R., http://www.bioinf.org.uk/software/profit/).

Coiled coils were modelled using the CoiledCoil class, with the from_parameters class method, using the parameter ranges described in Table 1. Optimization was performed over 50 generations, with 20 models in each, for a total of 1020 models including the parent generation.

**Table 1.** Parameter ranges used to model coiled coils

Oligomer state	Radius range (Å)	Pitch range (Å)	Interface angle range (°)
Dimer	3.5–5.5	50–350	−20 to 20
Trimer	5.0–7.0	50–350	−20 to 20
Tetramer	5.5–8.5	50–350	−20 to 20
Pentamer	6.5–9.5	50–350	−20 to 20

Collagen structures were also parameterized using the CoiledCoil class, with the tropocollagen class method. Hydroxyproline in the crystal structures was converted to proline to allow side-chain packing and structural alignment. The gross structural properties and therefore the parameterization of the fold are not affected by this change. Collagen was modelled with radii range of 1.5–5.5 Å; pitches in the range of 25–105 Å; unrestricted interface angles; a z-shift range for each helix of 0.0–6.2 Å staggered relative to each other; and a rotational offset -30° to 30° for each helix. Optimization was performed over 50 generations, with 30 models in each, for a total of 1530 models including the parent generation.

Models of Ankyrin-like peptides were built using the HelixPair class to generate the repeating unit and the Solenoid class to apply helical symmetry. The repeating unit was modelled with radii in the range of 0.0–6.0 Å, z-shifts in the range of -6.0 to 6.0 Å, unrestricted helical rotation, in-plane rotations in the range -45° to 5° and out-of-plane rotation range 90°–270°. Optimization was performed over 50 generations, with 50 models in each, for a total of 2550 models including the parent generation. The optimized repeating unit was used to model the solenoid with a radius range of 25.0–45.0 Å, rise per repeats in the range 2.0–18.0 Å, unrestricted twist range. The repeat unit was allowed unrestricted rotation during optimization. Optimization was performed over 100 generations, with 40 models in each, for a total of 4040 models including the parent generation.

The solenoid model of the TAL effector protein bound to DNA was built using the same base method described above, however the Solenoid class was given radii in the range 10.0–30.0 Å, rise per repeat values in the range 2.0–18.0 Å, unrestricted twist range. The repeat unit was allowed unrestricted rotation during optimization. Optimization was performed over 50 generations, with 20 models in each, for a total of 2040 models including the parent generation. The model of DNA was built using the DNADuplex class, and manually aligned, using tools included in ISAMBARD, with the solenoid to match the phase of the DNA and protein model. The final model was aligned with the experimentally determined structure, using ProFit, based solely on the protein region.

#### 2.2.1 RMSD_100_

In order to compare the quality of fit across a range of individual protein structures of different sizes, we calculated the RSMD_100_ value (Carugo and Pongor, 2001) using the following equation:

$${RMSD}_{100}=\frac{RMSD}{1+\ln\left(\sqrt{\frac{N}{100}}\right)}$$

## 3 Results

### 3.1 Specifications in ISAMBARD accurately recreate natural structures using parametric models

We tested our generalized parametric modelling in ISAMBARD by rebuilding a range of natural structures. The protein folds selected were α-helical coiled coils, collagen triple helices and Ankyrin-like repeats, as these are readily parameterizable and are of interest to the protein design and broader communities (Huang *et al.*, 2014; Jalan *et al.*, 2014; Parmeggiani *et al.*, 2015; Plückthun, 2015; Thomson *et al.*, 2014). Figure 3 shows that each of these folds have been successfully captured in ISAMBARD through two specifications: CoiledCoil and Solenoid.

#### 3.1.1 Coiled coils

The Crick equations (Crick, 1953) provide a parametric description of α-helical coiled coils. Previously, these have been successfully implemented for model building and protein design (Grigoryan and Degrado, 2011; Harbury *et al.*, 1995, 1998; Huang *et al.*, 2014; Offer and Sessions, 1995; Rämisch *et al.*, 2015; Thomson *et al.*, 2014; Wood *et al.*, 2014). Coiled-coil modelling has been implemented differently in ISAMBARD, using a more-general approach where the mathematics describing secondary structure is separated from that that describes the overall quaternary structure. This is vital for the modularity and re-usability of the parameterizations, and allows a wide array of different protein folds to be described using the same fundamental tools. Distinct secondary structure types are defined using the same specifications at the Polymer level. The Assembly level is independent of the Polymer-level specification, and so can be applied to different secondary structures types to yield different protein folds. For example, the CoiledCoil specification is used to model both α-helical coiled coils and collagens (see below). To test if the CoiledCoil specification accurately generated the degrees of freedom observed in experimentally determined X-ray crystal structures of coiled coils, the following selection of parallel coiled-coil assemblies was recreated in ISAMBARD.

**Fig. 2.**
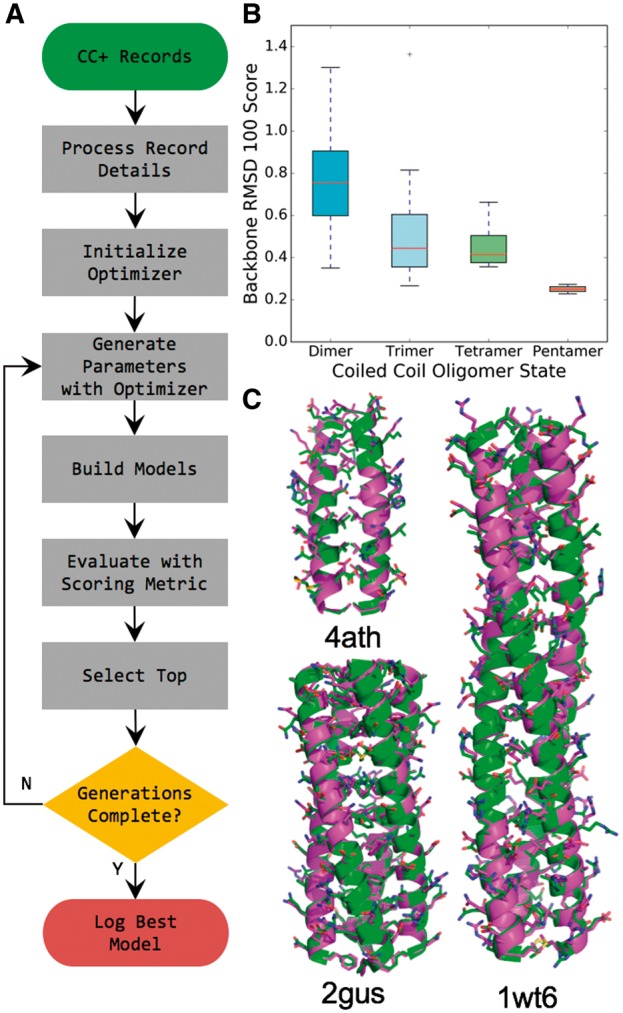
Crystal structures of coiled coils are recreated using parametric model building in ISAMBARD. (**A**) Model-building methodology for coiled coils employed to test the accuracy of ISAMBARD. The differential evolution optimizer was used with RMSD between the model and the experimental X-ray crystal structure as the scoring metric. (**B**) Box and whiskers plot of RMSD_100_ scores for non-redundant, dimers (cyan, n = 66), trimers (light blue, n = 41), tetramers (light green, n = 4) and pentamers (tan, n = 2) in CC+ (Testa *et al.* 2009), with more than a total of 44 residues. (**C**) Overlay of experimentally determined structure (green) with corresponding model (magenta), for a dimeric (4ath, RMSD = 0.48 Å), trimeric (1wt6, RMSD = 0.67 Å) and tetrameric (2gus, RMSD = 0.45 Å) coiled coil (Color version of this figure is available at *Bioinformatics* online.)

We searched the CC+ database for non-redundant, homomeric, parallel coiled coils in oligomer states ranging from 2 to 5 (Testa *et al.*, 2009), requiring that each structure contain at least 45 residues in order to apply the RMSD_100_ normalization function (Carugo and Pongor, 2001). This yielded 113 structures for rebuilding in ISAMBARD (Fig. 2).

The structural optimizer was initialized with the CoiledCoil specification, the amino-acid sequence and the oligomeric state of the structure being rebuilt as well as the three structural parameters (radius, pitch and φCα, Supplementary Fig. S2), which were optimized.

For each of the 113 structures, the values for each of the 3 parameters converged within 1020 models. The overall modelling accuracy was excellent, with a mean backbone RMSD of 0.64 Å (σ = 0.24 Å, n = 113). This shows that the parameterization contained in the CoiledCoil specification is sufficient to accurately model coiled coils, even though it describes the assembly using only 3 structural parameters, none of which need to be derived in the first instance from existing protein structures. This is an improvement over modelling with CCBuilder (Wood *et al.*, 2014), which gave an average backbone RMSD of 0.74 Å (σ = 0.45 Å, n = 113) for the same selection of coiled coils, and compares favourably with alternative coiled-coil modelling methodologies (Grigoryan and Degrado, 2011; Wood *et al.*, 2014). Thus, in our experience, the CoiledCoil specification in ISAMBARD is now the most accurate tool available for building parametric models of coiled coils.

#### 3.1.2 The collagen triple helix

The level of abstraction in the CoiledCoil specification means that it can be used directly to build models of collagen. This is because the gross geometry of collagen is similar to a coiled-coil trimer, although each component helix is a polyproline type-II helix rather than an α helix. An additional structural parameter, z-shift, is required to describe relative offset of the component helices along the long axis of the collagen molecule, which creates a leading and a lagging strand (Shoulders and Raines, 2009).

**Fig. 3.**
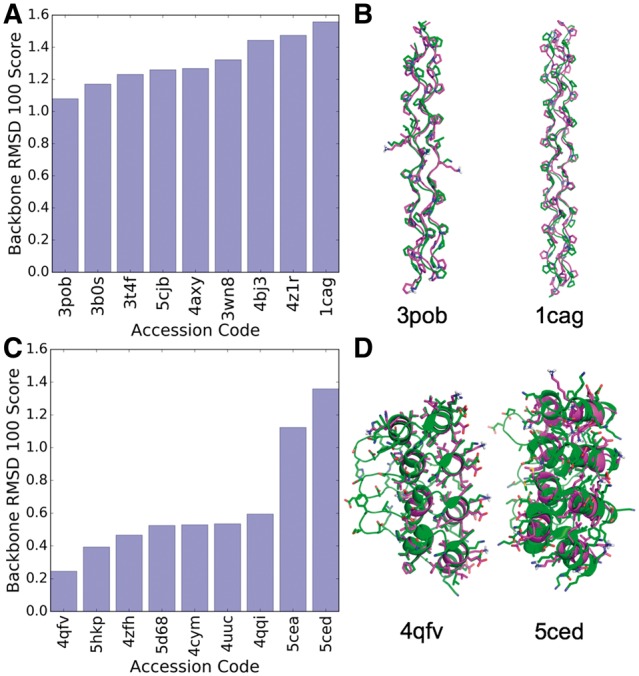
Models of natural structures built using parametric specifications in ISAMBARD. (**A**) RMSD_100_ scores for the backbone of rebuilt collagen and collagen-like peptides. (**B**) Overlay of models (magenta) and experimentally determined structure (green) for two representative collagen-like peptides, 3pob (left) and 1cag (right). (**C**) RMSD_100_ scores for the backbone of rebuilt Ankyrin-like proteins. (**D**) Overlay of models (magenta) and experimentally determined structure (green) for two representative Ankyrin-like proteins, 4qfv (left) and 5ced (right) (Color version of this figure is available at *Bioinformatics* online.)

A set of 9 representative, high-resolution crystal structures of collagen and collagen-like peptides was selected from the PDB and then their structures modelled using ISAMBARD. The parameterization accurately captured the backbone of the structures, with a mean backbone RMSD_100_ score of 1.31 Å (σ = 0.44 Å, n = 9) (Fig. 3, Supplementary Fig. S5, Supplementary Table S1). The difference between the best model and the worst was narrow, for example, RMSD_100_ score of 1.08 Å (3pob) and 1.57 Å (1cag).

The mean score was higher than for coiled-coil trimers, which had a mean value of 0.50 Å, (σ = 0.20 Å, n = 41). This is most likely due the overall flexibility of the collagen fold due to the broader energy well of the polyproline type-II helix (Kuster *et al.*, 2015). Further on this, the poorest areas of alignment were found at the *N* and *C* termini of the component polypeptides, where fraying of the X-ray crystal structures of the collagen fibres occurred. This is not observed to the same extent in coiled coils, and cannot easily be captured by parametric models. However, these models are still very accurate, and, to our knowledge, this is the only general method available for easily and rapidly generating atomistic models of the collagen triple helix backbone. The facile exploration of the collagen structural space through ISAMBARD may prove to be useful and complementary to existing methods of automated computational design of collagen fibres, which use a combination of discrete sequence-based models and geometric information from natural collagen fibres (Xu *et al.*, 2010, 2011).

#### 3.1.3 Ankyrin-like repeat proteins

Ankyrin-repeat proteins were selected as representative examples of α solenoids as there are several experimentally determined structures. Furthermore, recent designs of artificial Ankyrin-like repeat proteins, with a range of structural and functional diversity provide benchmark comparisons for our modelling (Boersma and Plückthun, 2011; Brunette *et al.*, 2015; Parmeggiani *et al.*, 2015; Plückthun, 2015). Models generated by ISAMBARD could form the basis of structural analysis of putative designs in attempts to create new Ankyrin-like repeat proteins with specific functions.

The models of α solenoids were built in two stages. Initially, the repeating unit of two short α helices was defined with the HelixPair specification and optimized for a given sequence, and then helical symmetry was applied with the Solenoid specification (Fig. 1).

Generally, in the Solenoid function, the repeating unit is built on a plane relative to a reference axis. The positions of the helices are described independently using 5 parameters: axis distance, z-shift, φCα, splay and off-plane rotation (Supplementary Fig. S3). As these parameters are independent, it is possible to create the same relative positions using different parameter values. Helical symmetry is applied to the repeating unit by defining the radius, twist per repeat, rise per repeat and the handedness of the solenoid. The repeating unit also has rotational freedom, needed to ensure that it remains oriented correctly relative to the helical axis (Supplementary Fig. S4).

Regular, parameterizable regions of a set of 9 representative high-resolution crystal structures of Ankyrin-like proteins were modelled using ISAMBARD (Fig. 3, Supplementary Fig. S6, Supplementary Table S2). The parameterization captured the conformation of the reference structures very effectively, with all RMSD_100_ scores below 1.5 Å, comparing favourably with the collagen-like peptides. Indeed, for 7 of the 9 structures, the RMSD_100_ was lower than 0.64 Å, the mean score for coiled coils.

This specification is the most complex of all those discussed herein, and required 7 parameters in total, compared to 3 for the parallel coiled coils and 4 for the collagen triple helix. Despite this, the models minimized in a similar time frame (4040 models, ≈ 10 minutes on a single core of a desktop computer). This demonstration of the quality of the differential evolution optimizer is certainly encouraging for modellers of even more-complicated folds and/or broader classes of protein folds.

Loops are crucial for the function of Ankyrin-like repeat proteins, and while it is not possible to model these regions parametrically, there are tools included in ISAMBARD, such as TAPolypeptide, that allow these to be modelled explicitly, by specifying a list of backbone torsion angles. Furthermore, once the backbone has been generated, the loop regions could be added to the model using one of a range of existing methods (de Bakker *et al.*, 2003; Bender *et al.*, 2016; Choi and Deane, 2009; Fiser *et al.*, 2000).

### 3.2 Different elements can be combined to generate complex models

**Fig. 4.**
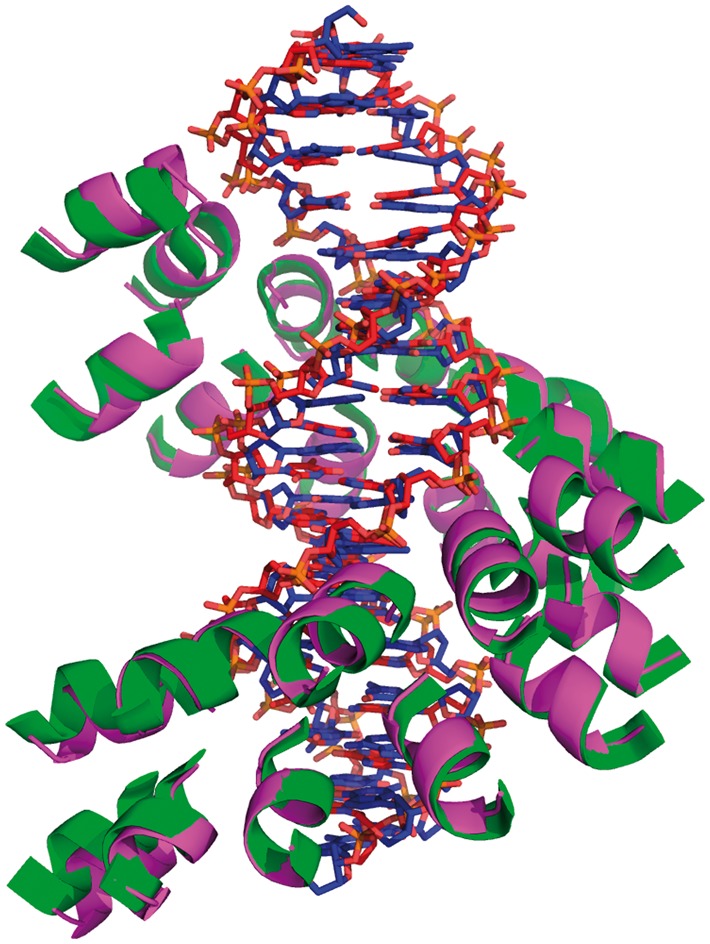
Crystal structure and ISAMBARD model of a TAL effector protein bound to DNA. Experimentally determined structure (3v6t, green and red) overlaid with a model (magenta and blue) created entirely using the ISAMBARD framework. Backbone RMSD = 1.03 Å (RMSD_100_ = 0.79 Å) (Color version of this figure is available at *Bioinformatics* online.)

Whilst ISAMBARD has been developed for parametric modelling of protein structures, most of its tools have been made as general as possible to enable their application to other biomolecules. To demonstrate this, we developed a straightforward specification for building parametric models of DNA, and used this in combination with the Solenoid specification to generate a model of a TAL effector bound to a DNA duplex. We used the rebuilding protocol to construct a model that recreates a known crystal structure (3v6t; Fig. 4).

The TAL-effector protein was constructed first, using the optimal parameter values for the Solenoid specification. With the protein model in hand, a DNA duplex was constructed using the DNADuplex specification (Fig. 1), which builds a DNA duplex based on sequences of its strands. The final model was created by rotating and translating the DNA object to bring it into phase with the TAL-effector (TALE) model using tools for geometric manipulation included in the ISAMBARD package (and built into BaseAmpal). The overall alignment of the parameterizable protein region of the TALE in Figure 4 with its model has a backbone RMSD of 1.03 Å (RMSD_100_ = 0.79 Å).

## 4 Conclusion

We have described ISAMBARD, a framework that provides a generalized approach to *in* silico parametric design and optimization of *de novo* biomolecular structure. We have shown that parametric modelling of proteins is an effective way to reduce the overall structural space that would otherwise prevent atomistic modelling, or at least make it a lengthy process for users. Even for models that require a relatively large number of parameters, as in the case of the solenoid proteins, it is possible to optimize the structure readily using the metaheuristics methods build into ISAMBARD.

The generic design of tools in ISAMBARD allows users to define their own parameterizations that are either completely novel, or composites of existing parameterizations. This focus on modularity makes it readily adaptable and extendable by the user. This ethos has been applied at all levels of the software design, enabling any user familiar with the project to extend and contribute to the code base. Indeed, we have benefitted from the modular approach: due to the model building generality, most of the tools required to model the collagen triple helix and α solenoids already existed in ISAMBARD before efforts began to parameterize these folds.

Currently, specifications are defined manually and then explored using automated optimization strategies. However, it is possible that these parametric models could be determined automatically, and we anticipate that future versions will have features to do this using machine learning strategies trained on structural data gathered using the analysis tools in ISAMBARD.

Our approach is complementary to other design and modelling suites, such as Rosetta and Modeller (Chaudhury *et al.*, 2010; Das and Baker, 2008; Eswar *et al.*, 2006). We envisage that powerful protein-design pipelines could be generated by combining ISAMBARD with these packages along with other tools for atomistic simulation such as OpenMM (Eastman *et al.*, 2013). Indeed, this would be facilitated by the availability of Python-based front-ends for these software suites.

More generally, the parameterized fold is not required to have any basis in a naturally observed protein fold. Thus, while most state-of-the-art protein design packages require some element of information from natural structures, ISAMBARD provides a starting point for going into the ‘dark matter of protein fold space’ (Taylor *et al.*, 2009; Woolfson *et al.*, 2015).

## Supplementary Material

Supplementary Video

Supplementary Data
