# Supplementary material for: ISAMBARD: an open-source computational environment for biomolecular analysis, modelling and design
Source: Bioinformatics. 2017 Jun 5;33(19):3043–50. doi: 10.1093/bioinformatics/btx352 (PMC5870769; doi:10.1093/bioinformatics/btx352)
Supplement: Supplementary Data [file bioinf-2017-0207_isambard_wood_si_btx352.docx]

**SUPPLEMENTARY INFORMATION**

(Supplementary Figure 1. Parameterisation Video)

**
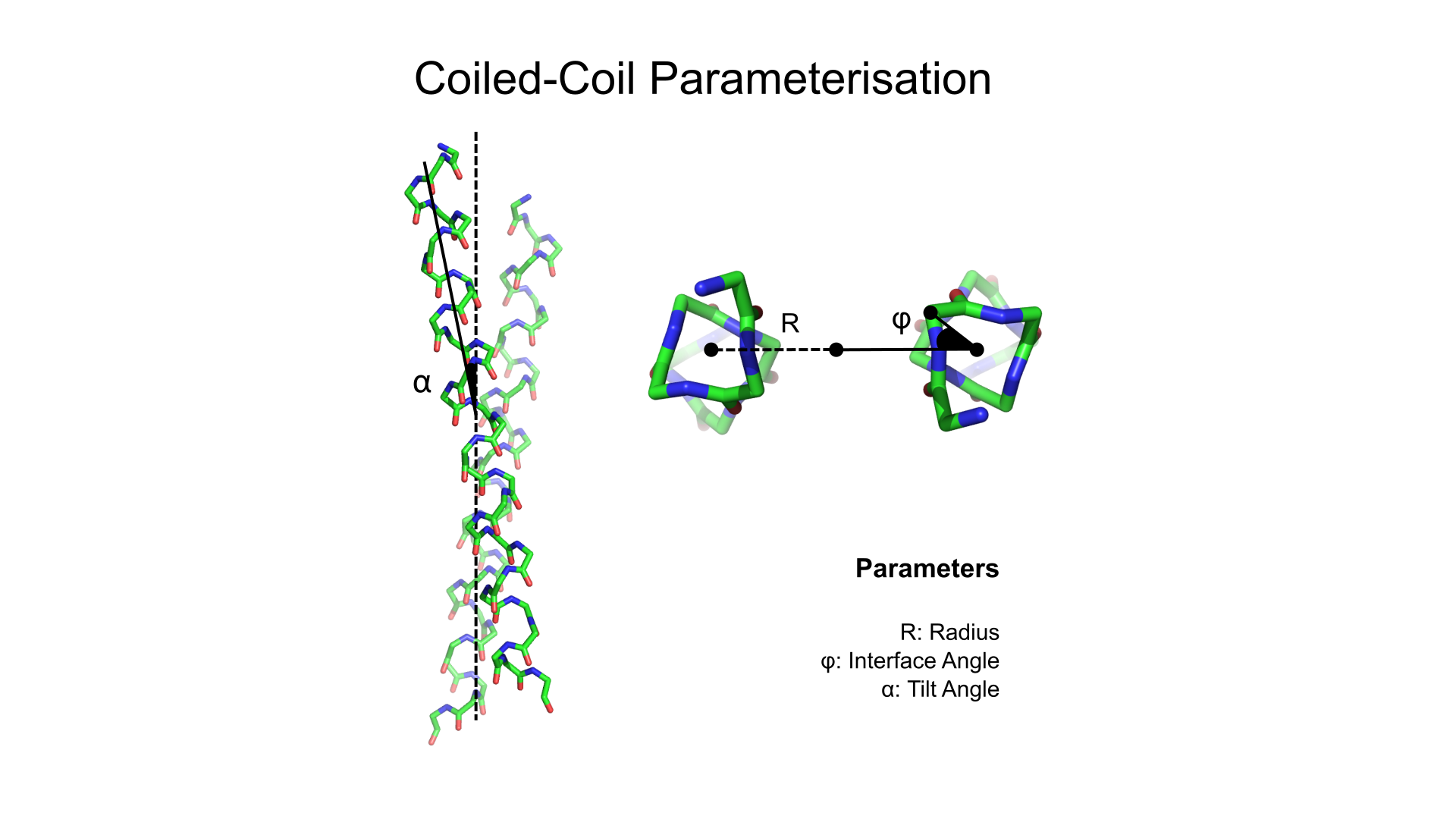
**

Supplementary Figure 2.

The major parameters used for the coiled-coil parameterisation contained in the CoiledCoil specification.


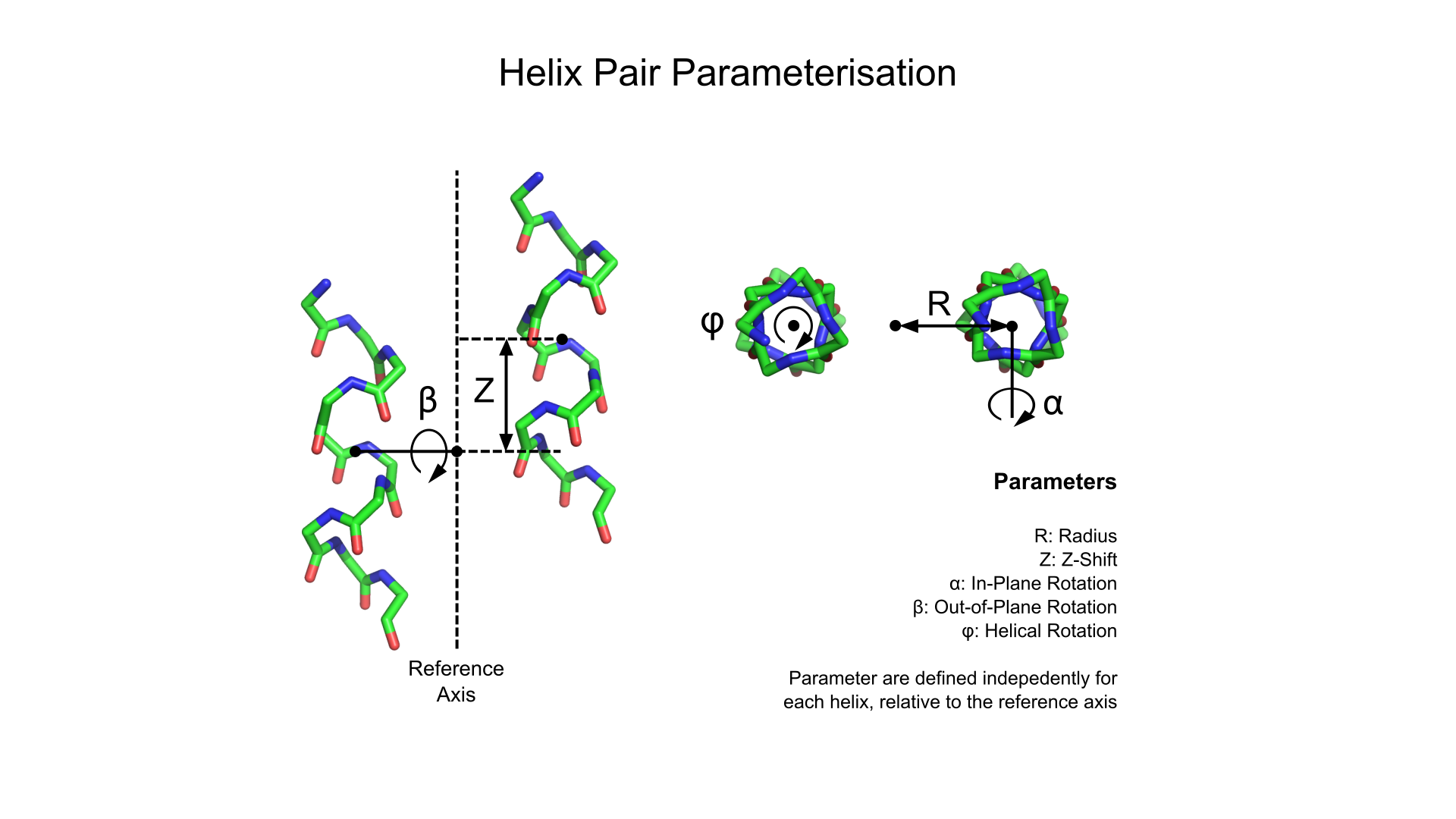


Supplementary Figure 3.

The parameters used for the “Helix Pair” parameterisation contained in the HelixPair specification. HelixPair is used to create the repeating unit of the solenoid models.


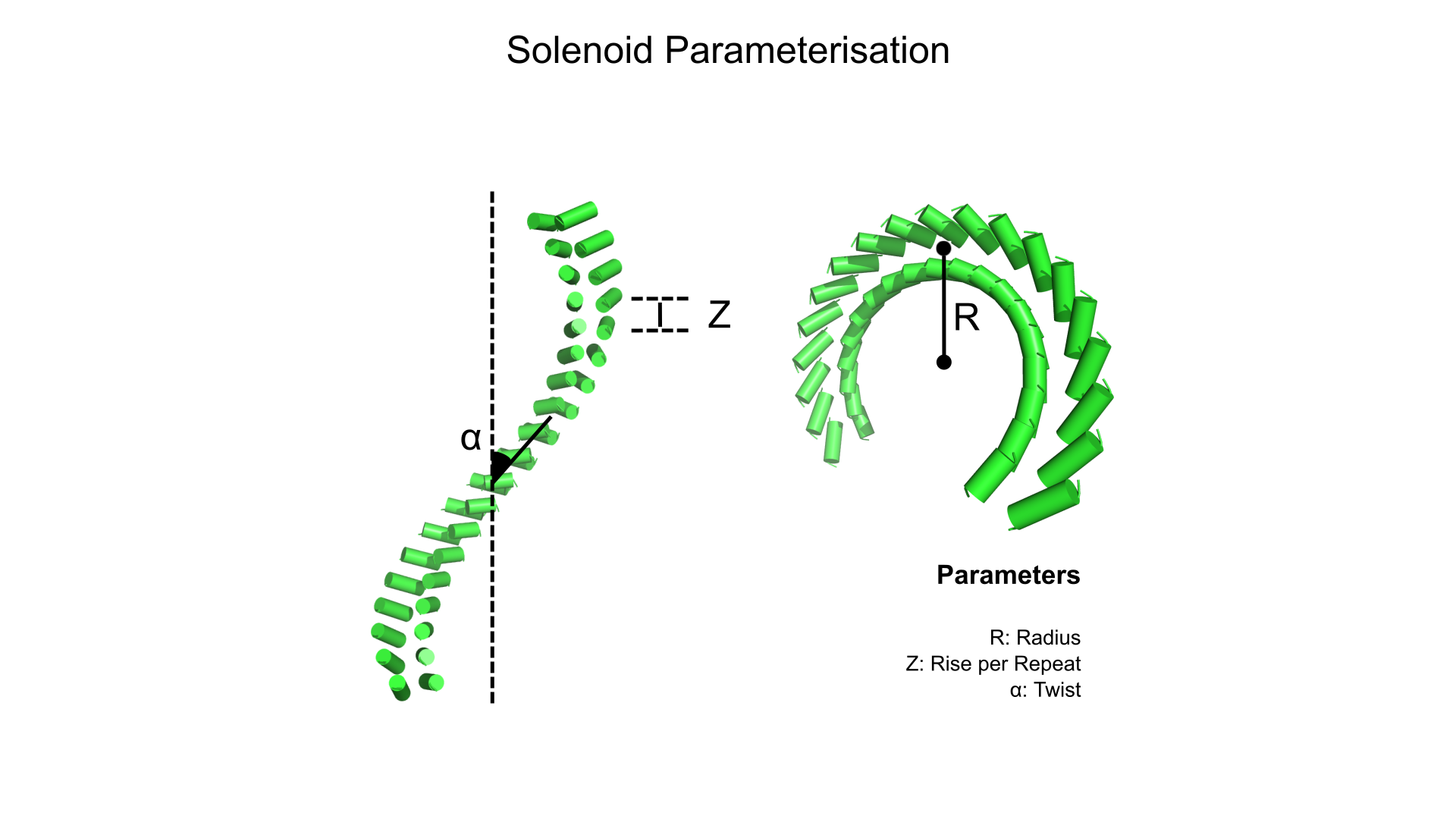


Supplementary Figure 4.

The parameters used for the solenoid parameterisation contained in the Solenoid specification. The repeating unit in this case is a HelixPair object.

**
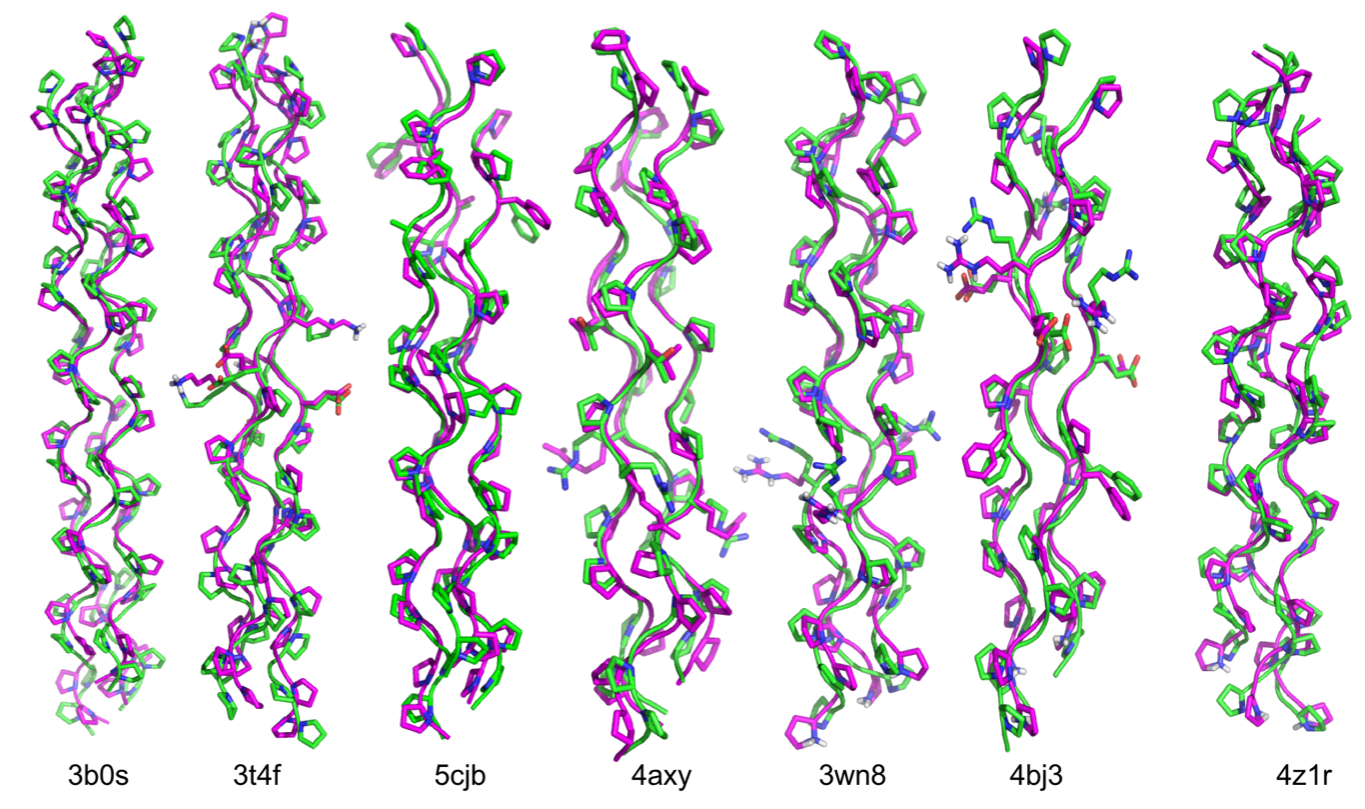
**

Supplementary Figure 5.

Models of natural collagen structures built using the CoiledCoil specification in ISAMBARD. Overlay of models (magenta) and experimentally determined structure (green) for seven collagen-like peptides. The PDB accession code is given below each model.


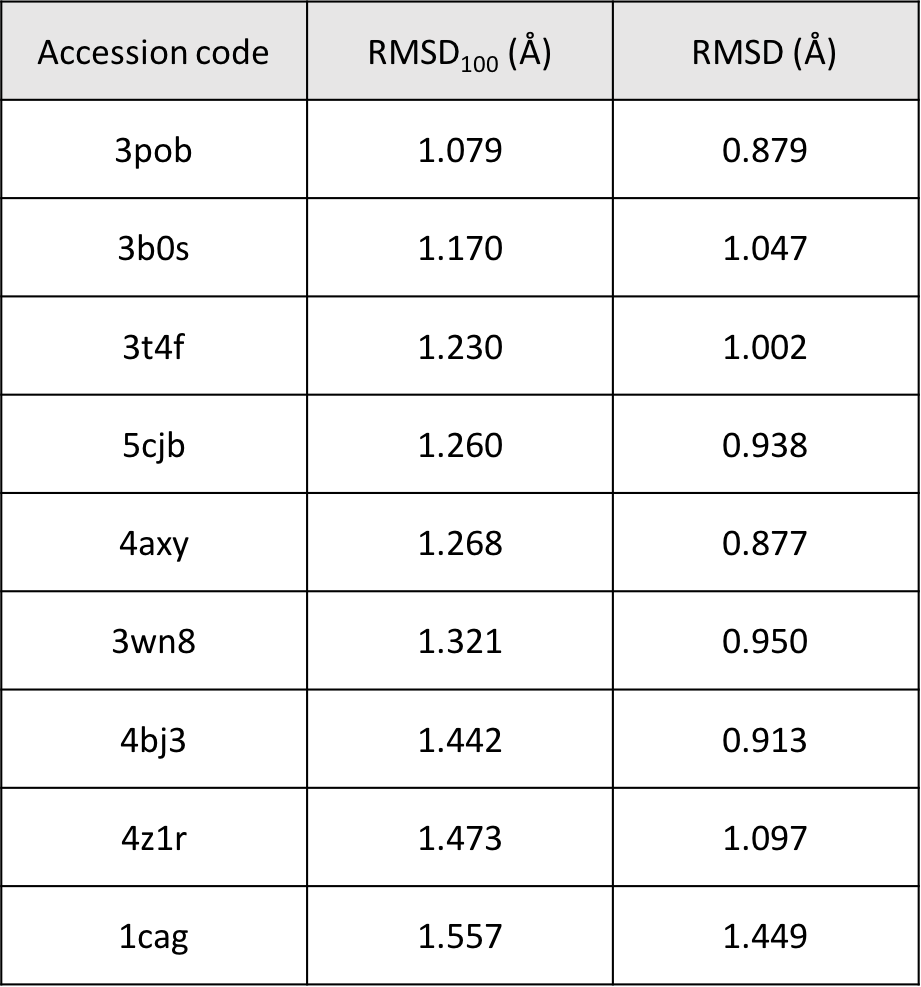


Supplementary Table 1.

The backbone RMSD_100_ and RMSD between the model and the experimentally determined structure is given for each of the collagen-like peptides modelled.


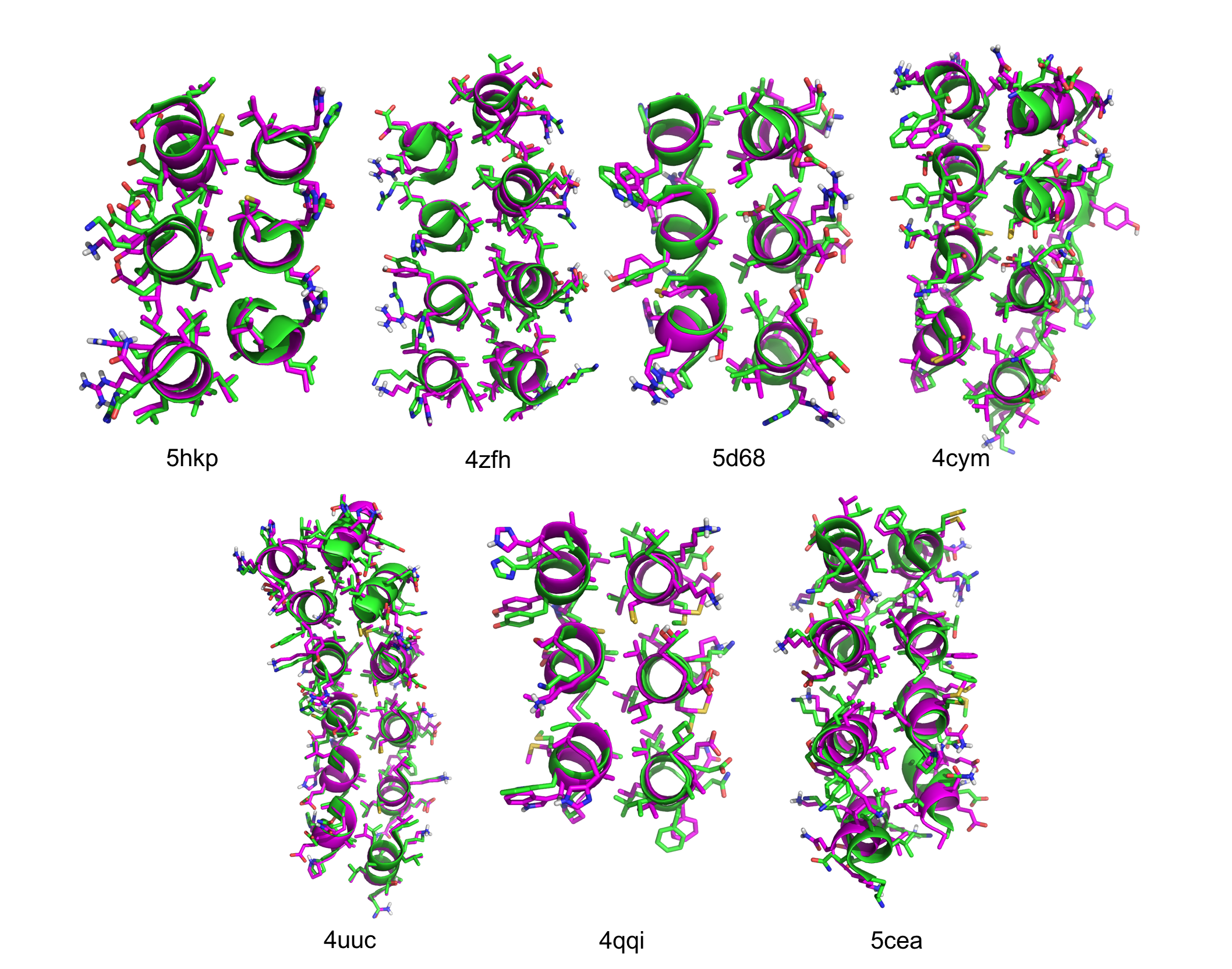


Supplementary Figure 6.

Models of natural Ankyrin-like proteins built using the Solenoid specification in ISAMBARD. Overlay of models (magenta) and experimentally determined structure (green) for the parameterised regions of seven Ankyrin-like proteins. The PDB accession code is given below each model.


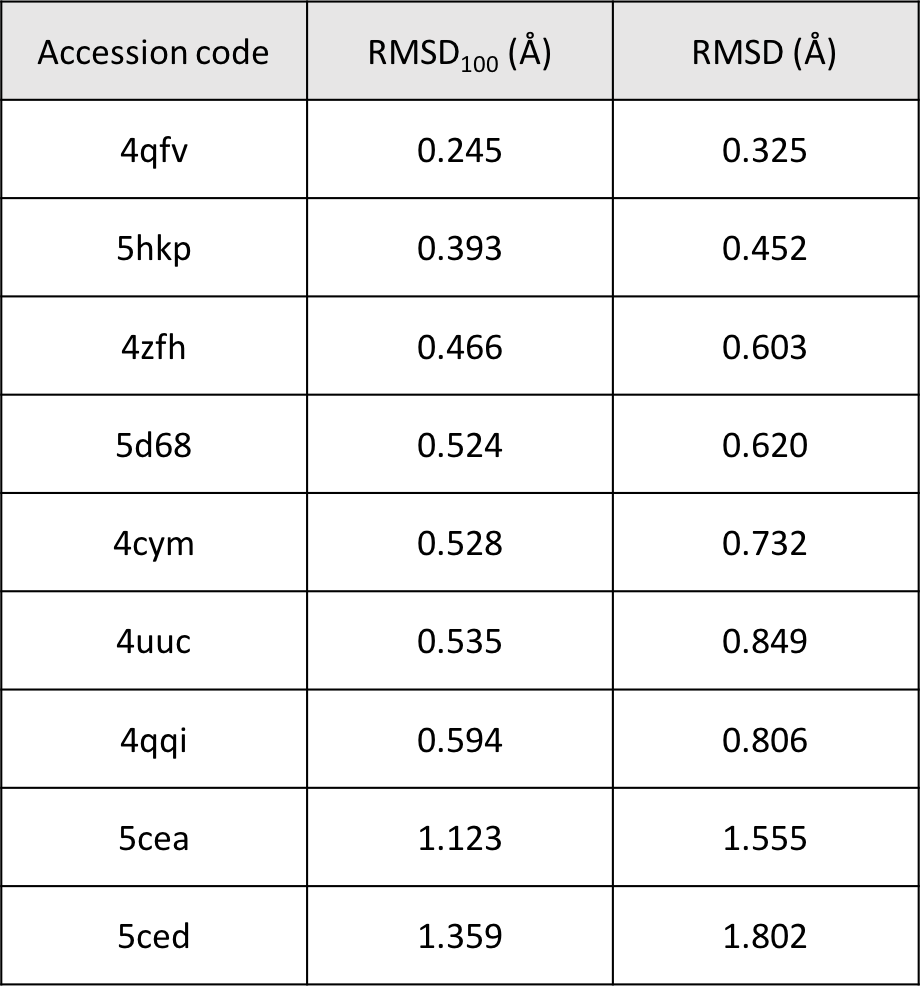


Supplementary Table 2.

The backbone RMSD_100_ and RMSD between the model and the experimentally determined structure is given for each of the Ankyrin-like peptides modelled.
